# Sympathetic Ophthalmia

**Published:** 1899-09-30

**Authors:** 


### SYMPATHETIC OPHTHALMIA.

In opening a discussion at the recent meeting of the British Medical Association on the Pathological Significance of Sympathetic Irritation and its Connection, if any, with Sympathetic Ophthalmia, Mr. Richardson Cross reaffirmed the broad doctrine, which in late years has been somewhat tampered with, that a damaged and useless eye should be removed. Of late years a distinct separation has been made between the cases of sympathetic inflammation and those of irritation or neurosis, the separation depending almost entirely upon the character of the symptoms which are present in the sympathising eye. These can be classed under two headings, as functional and inflammatory. The functional subjective disturbances typical of sympathetic irritation are weariness of the eye and disinclination for use, uneasiness, tenderness, or pain in the eye or orbital region; blepharospasm, lachrymation and hyperæmia of the conjunctiva, photophobia, muscæ, and photopsia; dizziness or fogginess of sight; obscurations of longer or shorter duration; transient defects in the visual field, or even temporary blindness; spasm or weakness of accommodation, or abnormal pupil action. A slight redness or haze of the optic papilla may be present, but careful examination of the eye fails to detect any signs of ocular inflammation. In "ophthalmitis," however, there are objective symptoms of definite inflammation which early implicates the uveal tissues. Impaired function of the pupil is accompanied by iritis, keratitis punctata, and ciliary congestion. The irido-cyclitis, usually of a mild, serous type, and at first causing the patient but little discomfort, insidiously implicates the whole uvea, and gives rise to synechia of the pupil and to vitreous opacities. Although the purely "functional" symptoms of irritation compared with the definite "organic" defects present in sympathetic ophthalmitis render the diagnosis between them easy in typical cases, clinically the two sometimes overlap one another, and cases occur in which the symptoms suggest that an irritated eye is being threatened with the more serious form of mischief. It must not be forgotten that the period of interval between the injury and the occurrence of the disturbance in the opposite eye may vary extremely. Both in inflammation and in irritation it may be very short or very long, so long, in fact, that one cannot feel absolutely safe after any period. It should, however, be stated that definite uncomplicated irritation is much more likely than inflammation to occur after a prolonged interval. As explaining the origin of the two conditions, the theory that the one is a reflex neurosis, and the other a migratory ophthalmia or septic uveitis, creeping round in some manner from the injured eye, has done good service as a working hypothesis, although it remains unproven. In regard to the relations between the two sets of cases it is to be noted that the course of sympathetic irritation may be that it may soon cease, or that it may recur from time to time for years without any further complications, or may be relieved by removal of the exciting eye, or that it may eventually be succeeded by an ophthalmitis. This is a most important point, and in view of the fact that a seriously damaged eyeball is prejudicial to its fellow and may predispose it to various discomforts if not diseases, while a shrunken globe interferes with the wearing of a glass eye and may at any time become a source of danger, the rule is laid down that an eye that is neither comely nor useful, and is in any degree a possible source of mischief to its fellow, should be enucleated.

